# Air Quality Implications of Using Ammonia as a Renewable Fuel: How Low Can NO_*x*_ Emissions Go?

**DOI:** 10.1021/acsenergylett.3c01256

**Published:** 2023-09-27

**Authors:** Srujan Gubbi, Renee Cole, Benjamin Emerson, David Noble, Robert Steele, Wenting Sun, Timothy Lieuwen

**Affiliations:** † School of Aerospace Engineering, Georgia Institute of Technology, Atlanta, Georgia 30332, United States; ‡ EPRI, Charlotte, North Carolina 28262, United States

## Abstract

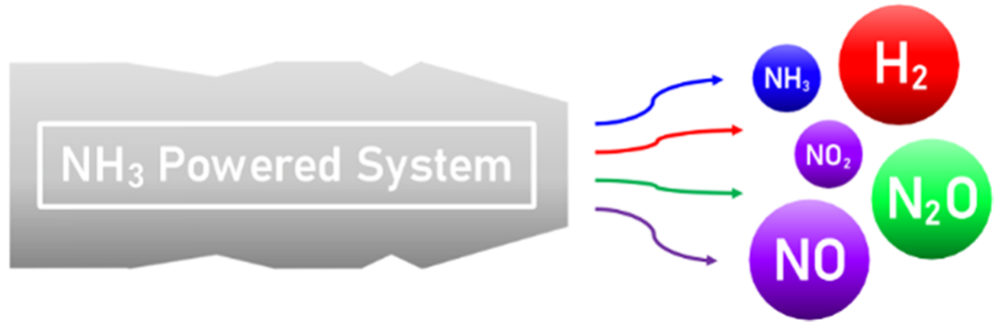

In addition to their lifecycle carbon emissions, another important issue with decarbonized energy pathways is their air quality, water, or land use implications. This paper considers the air quality issue for ammonia combustion. When directly combusting ammonia, reactions of its N atom with atmospheric oxygen lead to NO_*x*_ emissions that are O(10^3^) ppm, 2 orders of magnitude higher than EPA limits or the amount emitted by current natural-gas-fired technologies. In order to provide guidance to policymakers and technologists on what is fundamentally possible, this Perspective analyzes the fundamental minimum NO_*x*_ emissions that can be produced from ammonia combustion. The analysis shows that it is possible to achieve quite low NO_*x*_ emission levels of O(10) ppm, but these designs differ markedly from those used in today’s lean, premixed combustion systems.

A variety of net-zero energy pathways are being explored to meet the needs of future energy systems that are low-cost, resilient, and equitable. In addition to their lifecycle carbon emissions, another important issue with candidate decarbonized energy pathways is their air quality, water, and land use implications. These issues arise from an array of sources, including mining of raw materials, water utilization or discharges, emissions into the ambient air, and end-of-life disposal. Each of these issues has profound implications for achieving an energy transition that minimizes negative societal impacts. This paper considers the air quality implications of ammonia utilization through direct combustion, such as in an electric power plant or industrial heater.

Ammonia (NH_3_) has a deep foundation in the fertilizer industry, and a vast ammonia infrastructure already exists for its production and transportation.^1^ With the need for deep decarbonization of energy, transportation, and agriculture, ammonia is being actively investigated as a carbon-free energy carrier, both as a means for storing intermittent renewable energy sources and as a means of facilitating the transport of hydrogen. A recent report^2^ also concluded that, for large-scale, long-duration, transportable energy storage, ammonia and hydrogen are the two most promising solutions. Ammonia may be preferable to pure hydrogen for several applications, particularly because it is more readily transported as a liquid and has a large existing industrial base for production and transport to draw from.^3^ The International Energy Agency (IEA)^4^ forecasts that hydrogen-based fuels (including ammonia) should achieve approximately 30% of transportation fuel by 2050 to hit zero emissions.

Recent research efforts on the application of ammonia as fuel have been summarized by Valera-Medina et al.^3^ and Kobayashi et al.,^1^ including assessments of its combustion characteristics, such as flammability range, flame temperature, and flame speed. Ammonia can be used directly in end-use applications or cracked back to a H_2_/N_2_ blend. While direct application of ammonia has a number of cost and efficiency benefits, the presence of fuel-bound nitrogen can potentially lead to significantly more NO_*x*_ emissions during combustion than when burning it as H_2_/N_2_.

A sampling of measured emission values in the literature^5−15^ shows NO_*x*_ emissions from ammonia combustion that are ∼200–5000 ppm, which is 1 or 2 orders of magnitude higher than current industry benchmarks for natural gas combustion or existing U.S. Environmental Protection Agency (EPA) regulations (typical values are 10–30 ppm^16^). For example, lean premixed combustion technologies developed for low NO_*x*_ combustion of hydrocarbons commonly achieve NO_*x*_ emissions from 1 to 30 ppm, depending upon firing temperature.^17^

In this Perspective, we address the minimum theoretically achievable NO_*x*_ emissions from direct ammonia combustion. It aims to answer two important questions: (1) What are the fundamental limits on how low NO_*x*_ emissions can be from ammonia combustion, and (2) how should a combustion/reactor system be configured to approach these limiting values? In other words, we do not model a particular system’s behavior but determine the fundamental achievable value without postcombustion gas cleanup. The purpose of this analysis is to guide policymakers on what is possible, to allow technologists to contextualize how emissions of a given system compare to this fundamental limit, and to provide insights on architectures that move toward these limiting values.

## Theoretical Analysis and Numerical Framework

There are essentially no optimized, commercial ammonia combustors that exist today, but demonstrations are taking place and there is a rapidly developing literature evaluating different combustion strategies both to address emissions and to achieve acceptable operational limits.^5,11,13,18^ This developing literature is a key motivator for the present Perspective, which aims to contextualize these measurements relative to fundamental limits. Staged combustion systems are commonly used today for enhancing turn-down and reducing NO_*x*_. In systems utilizing fuels without molecularly bound nitrogen (such as hydrogen or natural gas), a secondary fuel stage (e.g., an “axial fuel stage”) is used to minimize NO_*x*_ emissions at elevated combustor exit temperatures.^17^ In these systems, a majority of the fuel is burned in a lean, premixed mode in the primary zone at a low temperature to avoid significant NO_*x*_ production. Then, if the desired combustor exit temperature is high enough to lead to significant NO_*x*_ formation, the remaining fuel is added in a secondary lean stage, where quick mixing is followed by combustion in order to minimize residence times at high temperature conditions, while still consuming all of the fuel.^17^ However, such a lean, premixed strategy for ammonia combustion leads to drastically higher NO_*x*_ emissions than non-nitrogen-containing fuels, as mentioned above and quantified later.

**Figure 1:**
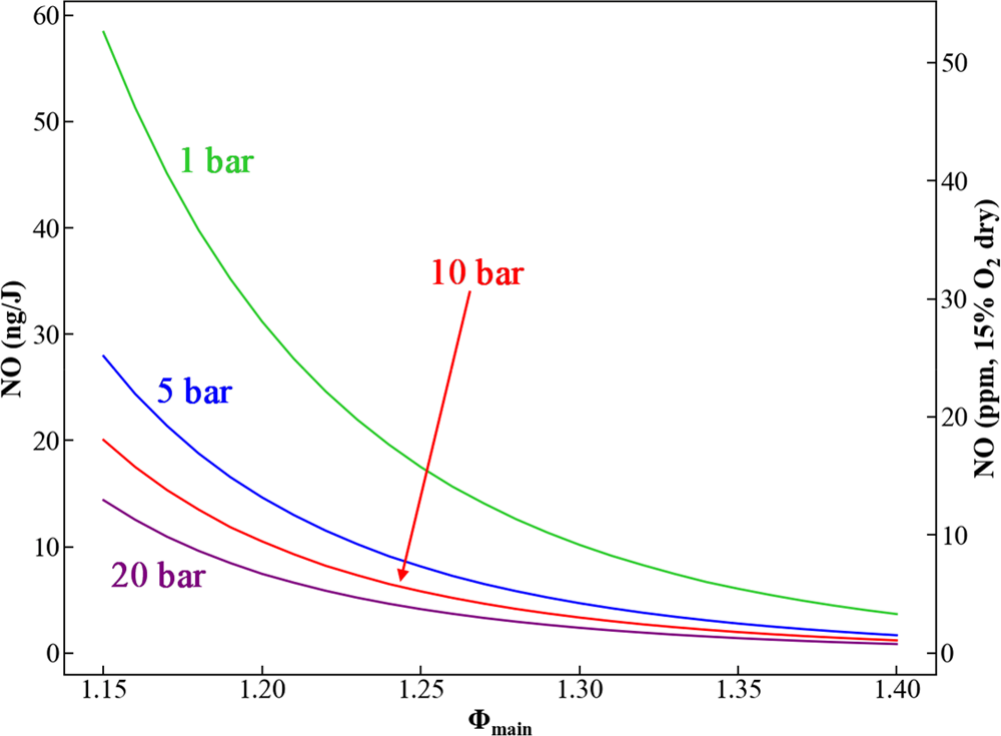
Equilibrium NO levels as a function of equivalence ratio and pressure for combustion of ammonia and air (initial ammonia temperature of 300 K and air temperature of 650 K).

Industry has developed limited NO_*x*_ control approaches in systems with nitrogen-containing fuels, such as liquid fuels and coal, by utilizing staged systems with a fuel-rich primary stage. This strategy is also relevant to ammonia combustion due to the fact that equilibrium NO_*x*_ emission from ammonia flames decreases sharply when the equivalence ratio of the ammonia/air mixture exceeds unity (see Figure 1). This leads to unburned fuel in the form of hydrogen as well as diatomic N_2_.^1^ Kinetically, this occurs via the abundant NH_2_ radical pool produced in rich flames that subsequently react with NO and drive it toward its equilibrium value. This N_2_/H_2_ (as well as other combustion products) can then be mixed with the remaining excess air under fuel lean conditions, which also minimizes NO_*x*_ formation rates if combustion occurs in a premixed condition. This concept is related to (but there also significant differences, as enumerated later) the Rich–Quench–Lean (RQL) staged combustion strategy, where a rich flame zone is followed by a lean flame zone to reduce NO_*x*_ levels from ammonia combustion.^14,15,18−21^ Such an RQL configuration leads to substantially less NO_*x*_ production than if the system were operated with a single stage at the same overall equivalence ratio, with reported NO_*x*_ emissions from calculations as low as 50 ppm.^22^ However, high H_2_, NH_3_, or N_2_O emissions can also occur if not fully optimized.^11^ In summary, basic reasoning associated with equilibrium tendencies of rich ammonia combustion and thermal NO_*x*_ production of lean mixtures indicates that a rich stage followed by a lean stage combustion strategy is the desired means to minimize the NO_*x*_ emissions from ammonia combustion. However, what is not clear is what the fundamental limits are—i.e., what the fundamental “floor” on NO_*x*_ emissions is for ammonia combustion based on chemical and physical considerations.

In this Perspective, we establish the theoretical minimum NO_*x*_ achievable for a gas-phase, constant-pressure system using ammonia. As such, it is relevant for a host of applications including gas turbines, boilers, heaters, and furnaces. In other words, the objective of this work is not to simulate a given combustion system, but rather the fundamental minima that can be achieved via technological development.

**Figure 2:**
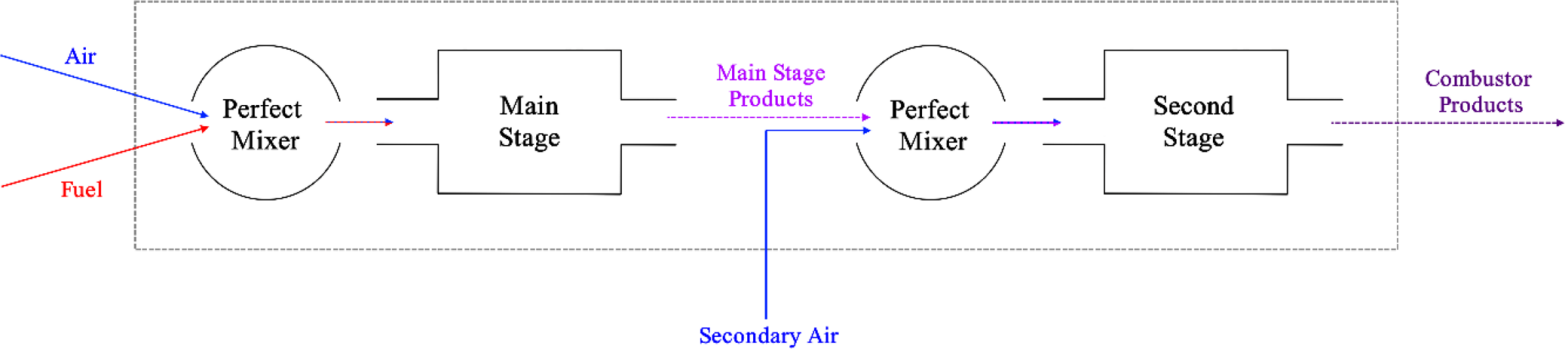
Schematic of the staged combustor.

While detailed modeling approaches can be found elsewhere,^23,24^ the rest of this section briefly summarizes the analysis approach. The basic schematic is illustrated in Figure 2. Fuel and air in the main stage are mixed adiabatically and then combusted in the fuel-rich main stage. The combustion products of this main stage, which includes significant levels of H_2_, are then adiabatically mixed with the secondary air and combusted in the secondary stage. More details on the reduced order modeling approach can be found in the Supporting Information. This system is optimized subject to three constraints: (1) a given global stoichiometry value, Φ_global_ (or, equivalently, a given combustor product temperature, which would be the prescribed parameter for a gas turbine application), (2) overall residence time, τ_global_ (typical values in modern gas turbines range from 5 to 20 ms, depending upon type^17^), and (3) exhaust emissions. With respect to this third constraint, we constrained exit H_2_ values to 1.25 times its equilibrium value at the combustor exit temperature (the exact value of this multiple generally has a negligible impact on results). Note that NH_3_ and N_2_O levels themselves are in the parts per billion (ppb) level because of rapid decomposition at flame temperatures.^25^ Indeed, if NH_3_ or N_2_O levels were used as the constraint, it is very possible to have significant levels of unburned fuel in the form of H_2_, while having NH_3_ or N_2_O emissions in ppb levels. Sufficient residence time is required in the second stage to oxidize H_2_, even while such a condition promotes NO_*x*_ formation via the Zeldovich mechanism and other routes. As such, the optimized configuration essentially comes down to balancing the stoichiometries of the main (Φ_main_) and secondary (Φ_sec_) stages, as well as the residence times of the main and secondary stages (τ_main_ and τ_sec_, respectively, where the prescribed τ_global_ = τ_main_ + τ_sec_) that minimizes exit NO_*x*_ values. Generally speaking, it is favorable to keep τ_sec_ low to limit NO_*x*_; however, sufficient time is needed to oxidize H_2_, which is why the third constraint is such an important one.

In the next section, results are displayed only for NO as it is the dominant species contributing to NO_*x*_ emissions in ammonia combustion at ideal conditions; NO_2_ and N_2_O values are several orders of magnitude lower. All emissions are specified in nanograms per Joule of energy input (ng/J) and parts per million (15% O_2_ dry).

## Analysis of NO Production Sources

**Figure 3:**
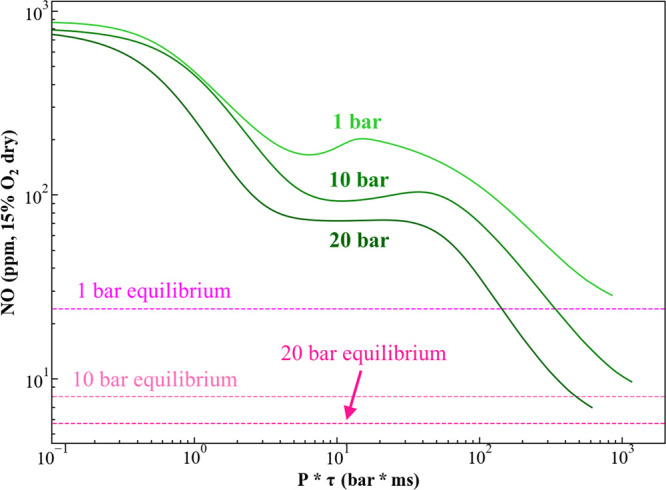
Scaled residence time sensitivity of NO evolution of a premixed ammonia/air flame at Φ = 1.22.

In order to understand the results, it is beneficial to examine the individual NO contributions between the main and secondary stages. Note that initial oxidation of NH_3_ leads to very high initial levels of NO (∼1000 ppm), followed by relaxation toward equilibrium. As such, it is helpful to further decompose main stage NO into an equilibrium contribution (the minimum possible NO from the main stage) and some extra amount of “unrelaxed” NO whose value varies with residence time. The resulting decomposition is as follows:
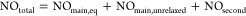
1The NO evolution for a rich ammonia flame is shown in Figure 3. Note that very long residence times are required for a rich NH_3_/air mixture to reach equilibrium, with this relaxation time and equilibrium value both dropping with pressure. In other words, increasing pressure has both a kinetic and a thermodynamic equilibrium effect, both acting in the same direction to decrease NO. Also, note that we have scaled the *x*-axis with pressure to readily illustrate different pressure results together. Note that these residence times required to reach equilibrium are substantially higher than those in modern gas turbine combustors (where typical values are in the 5–20 ms range^17^), where low residence times are desirable to minimize Zeldovich NO_*x*_ formation. Although the residence times required to reach equilibrium may not be feasible for currently fielded combustors, this shows that dedicated ammonia combustor designs will want to maximize residence time as opposed to minimizing it as is done in current combustor technologies.

Referring back to Figure 1, an equilibrium calculation is shown that quantifies NO_main,eq_ as a function of equivalence ratio and pressure. High pressure and richer main stage conditions are favored for lower NO emission, as expected. These calculations suggest that if an ammonia combustor is designed that allows the main stage to reach equilibrium and minimizes NO production in the secondary stage, sub-10 ppm NO emissions can be achieved, values that are well under current EPA limits.

## Optimum Configurations and Minimum NO

### Combustor Firing Temperature Sensitivities

**Figure 4:**
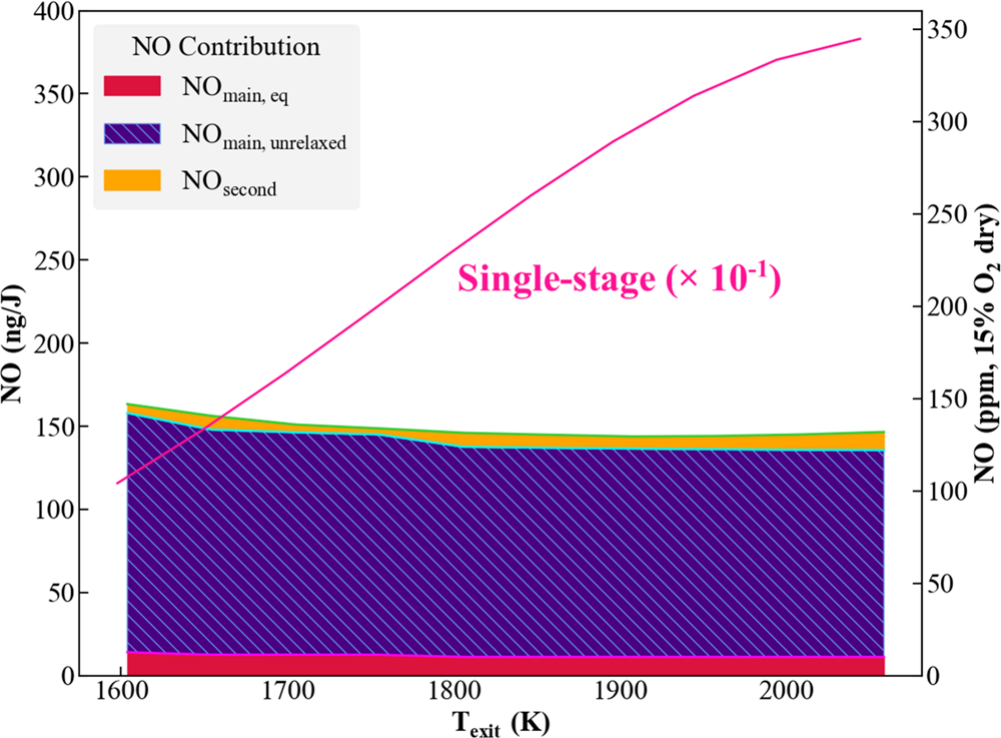
Minimum NO levels as a function of exit temperature (*P* = 2 bar). The solid pink line denotes the NO emissions that would be produced if NH_3_ were combusted in a single-stage, lean, premixed system. Note that this line divides the actual value by 10, so actual emissions are in the 1000–3500 ppm range.

Figure 4 plots minimum NO_*x*_ levels over a range of firing temperatures from 1600 to 2050 K for a 20 ms combustor residence time at 2 bar. As quantified later, the residence time certainly can be extended in simulations to achieve even lower emission levels. We limited the residence time to 20 ms in this simulation, as this is a typical value for gas turbines. The different NO contributions are designated by the shaded areas, also shown in Figure 4. The bottom shaded region (NO_main,eq_) represents the NO level if the main stage residence time was extended to allow it to reach equilibrium. Results indicate that higher emissions occur at lower firing temperatures and NO emissions under 150 ng/J (135 ppm) are achievable at higher firing temperatures. The majority of contribution is from unrelaxed NO (NO_main,unrelaxed_) in the main stage, indicating that lower emissions can be achieved if the combustor residence time is increased to allow the system to achieve chemical equilibrium. This indicates that the rich stage must be followed by a “relaxation stage” before the lean combustion stage, a feature that is distinct from that in RQL designs for hydrocarbons. For comparison purposes, the solid pink line represents the NO emission if the ammonia/air mixture is consumed directly in a single-stage, premixed burner—the approach used in current low NO_*x*_ combustion designs. It can be clearly seen that the rich/lean staged combustion strategy enables an order of magnitude reduction in NO emissions. It also shows that lean, premixed combustion leads to higher NO emissions at elevated temperatures, the opposite dependence of the rich staged system.

### Combustor Pressure and Residence Time Sensitivities

As can be anticipated from the above discussion, pressure and residence time have significant influences on the minimum NO levels. From a practical point of view, the required pressure and residence times both have significant capital cost implications. Specifically, they influence the required pressure ratings for compression equipment and pressure vessel ratings for high temperature components. They also increase the size and length of these high-temperature components, as long residence times increase the overall size of the combustion system. At elevated pressure conditions, ammonia could exist in the liquid phase with a high enthalpy of evaporation, depending upon its temperature.

These results show that it is not fundamentally possible to achieve sub-100 ppm values at atmospheric pressure without residence times exceeding 100 ms; this implies that a host of atmospheric applications that are capable of sub-10 ppm NO_*x*_ emissions with natural gas today, such as water heaters, furnaces, and boilers, will require significant design changes to allow acceptable NO_*x*_ emissions.

**Figure 5:**
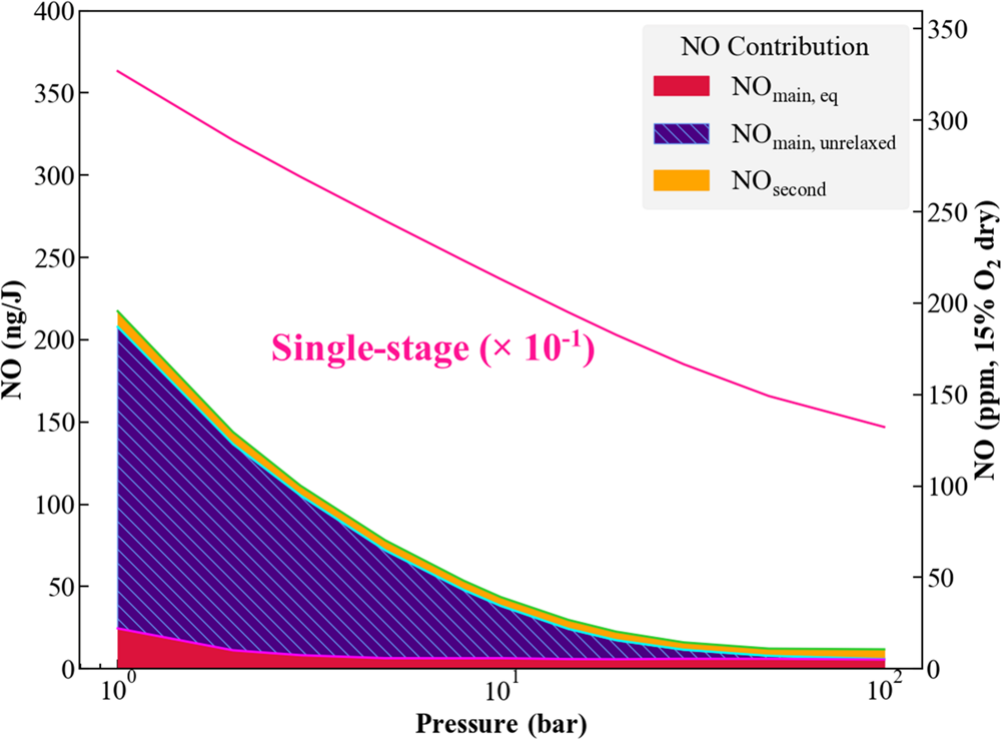
Minimum NO at various combustor pressures (*T*_exit_ = 1900 K, τ_global_ = 20 ms). The solid pink line denotes the NO emissions that would be produced if NH_3_ is combusted in a single-stage, lean, premixed system. Note that this line divides the actual value by 10, so actual emissions are in the 1300–3500 ppm range.

Consider further these pressure sensitivities at a fixed global residence time of 20 ms. Figure 5 presents results at the 1900 K combustor exit temperature. High pressure contributes to a faster approach toward equilibrium in the main stage, as noted previously, and this resultant decrease in the unrelaxed NO contribution is significant. For high pressure at these conditions, minimum NO emissions could be as low as 21 and 15 ppm at pressures of 20 and 30 bar, respectively. Noting the contribution from NO_unrelaxed_, this plot also shows that levels as low as 10 and 9 ppm are achievable at these two pressures with longer residence times. Finally, for comparison purposes, the solid line represents the much higher NO emissions that would result if the ammonia/air mixture is combusted directly in a single-stage combustor under the same conditions (exit temperature, pressure, and residence time). It can be clearly seen that the staged combustion strategy can reduce NO emissions by approximately 100 times at the 20 bar pressure condition. From Figure 5, it can also be observed that the sensitivity of absolute NO emissions on pressure starts to diminish above ∼20 bar. An engineering implication of this point is that designing systems to operate at approximately 20 bar can be quite effective, a useful result as this is very similar to the pressure condition where existing industrial gas turbine power generation systems operate. As such, this would imply that a changeout in the combustor would be needed, but not in larger cycle conditions (such as turbomachinery), if a power plant retrofit from natural gas to ammonia.

**Figure 6:**
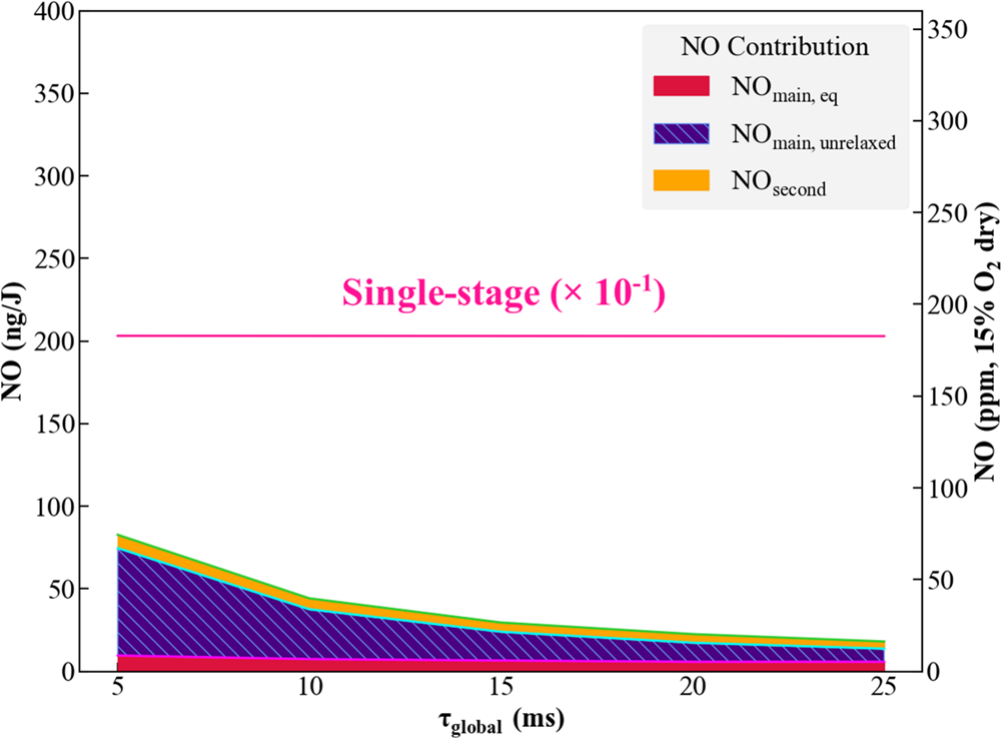
Minimum NO at various global residence times (*T*_exit_ = 1900 K and *P* = 20 bar). The solid pink line denotes the NO emissions that would be produced if NH_3_ is combusted in a single-stage, lean, premixed system. Note that this line divides the actual value by 10, so the actual emissions are about 1800 ppm.

Figure 6 plots residence time sensitivities at a fixed pressure of 20 bar and an exit temperature of 1900 K. As expected, the minimum achievable NO decreases, as the main stage unrelaxed NO contribution is lower for longer residence times. Further relaxation in the main stage is possible due to the longer residence times, and this effect is apparent in the shaded contribution areas in Figure 6.

A useful role in understanding these theoretical minimum values is contextualizing NO values reported from computations or measurements. For example, Rocha et al. reported a computational study with NO values as low as 50 ppm at Φ_main_ = 0.4, *P* = 20 bar with an inlet temperature of *T* = 500 K for an RQL configuration.^11^ Using these conditions, the theoretical minimum NO_*x*_ emissions at this condition is about 25 ppm, meaning that this combustor’s NO emissions are about twice the theoretical minimum.

To summarize, this work has developed values for minimum achievable NO emissions from the direct combustion of ammonia. These calculations show that EPA-compliant sub-30 ppm NO emissions levels are achievable with design conditions similar to conventional gas turbine conditions (e.g., 20 bar, 20 ms residence time). Even greater reductions are possible at higher pressures and longer residence times (both of which lead to higher capital costs). For example, 10 ppm NO values are achievable at 20 bar by increasing residence times to about 50 ms. Similarly, 10 ppm NO values are achievable at 20 ms at elevated pressure values of 100 bar. This work also demonstrates the opposing sensitivities of these results to lean, premixed designs running fuels that do not contain molecularly bound nitrogen, such as natural gas. In these cases, NO production is very sensitive to temperature (compared to the weak temperature sensitivity noted here) and increases with the combustor residence time.

## Supporting Information Available

Description of the reactor network model (PDF)
