# Supplementary material for: Air Quality Implications of Using Ammonia as a Renewable Fuel: How Low Can NOx Emissions Go?
Source: ACS Energy Lett. 2023 Sep 27;8(10):4421–6. doi: 10.1021/acsenergylett.3c01256 (PMC10580705; doi:10.1021/acsenergylett.3c01256)
Supplement: Supplementary file 1 — nz3c01256_si_001.pdf [file nz3c01256_si_001.pdf]

SUPPORTING INFORMATION

Air quality implications of using ammonia as a renewable fuel – How low can NO_x emissions go?

Srujan Gubbi[1], Renee Cole[1], Benjamin Emerson[1], David Noble[2], Robert Steele[2], Wenting Sun[1], Timothy Lieuwen[1]**

[1]School of Aerospace Engineering, Georgia Institute of Technology, Atlanta, GA 30332

[2]EPRI, Charlotte, NC 28262

AUTHOR INFORMATION

Corresponding Authors

*Wenting Sun: wenting.sun@aerospace.gatech.edu

*Tim Lieuwen: tim.lieuwen@aerospace.gatech.edu

Reactor Network Model Reduced order modeling by way of a chemical reaction network (CRN) was used in this study for the minimum NO_x calculation (see Fig. 2). The reduced order modeling approach allows the main stage and second stage to be split and modeled as separate reaction networks. The reactor network modeling assumes a perfect premixed, 1-D flame that consumes the reactants in each stage. Adiabatic, constant-pressure batch reactors were used to mix various streams of gas coming into each stage. For the main stage, streams of fuel and oxidizer were mixed, while for the second stage, the products from the main stage were mixed with secondary air. The mass flow rates for each stream were calculated based on the global and main stage equivalence ratios (Φ_{global} and Φ_{main}). The global equivalence ratio is specified, based upon the target combustor exit temperature.

The free flame model[26] outputs solutions in spatial coordinates, so numerical integration of axial velocities and distances between grid points was necessary to convert the solution to temporal coordinates. Although the flame zone generally starts at the same point in spatial coordinates, variations in flame speeds at different operating conditions cause variations in starting times for the flame. For this reason, peak NH_2 concentration was used to define the start of the flame zone. Therefore, time zero starts to count from where NH_2 peaks to ensure a consistent definition of residence time. As one of the first species in the ammonia-air reaction pathway[27], it is a useful baseline for referencing the rest of the system in temporal coordinates.

All calculations were done using Cantera[26], which is an open-source library that can simulate chemical kinetics problems. The kinetic model used in this study was developed by Mei et al. [28]. A few sample comparisons of the performance of different kinetic models and the selection of this kinetic model are provided in Fig. S1 and S2.

In the simulation, the fuel was pure ammonia, with a fixed temperature of 300 K. Oxidizer was synthetic air (79% N_2 and 21% O_2), with a preheat temperature of 650 K. Optimization was achieved at fixed values of Φ_{global} (overall equivalence ratio which controls combustor firing temperature), combustor pressure, and τ_{global} (total residence time) for a specific case. Each of these parameters was also individually varied to study their effects on minimum NO, which will be discussed in later sections.

Several kinetic models for ammonia combustion have been published, so a comparison was done to see which kinetic model would be most suitable for this study. Ignition delay times and laminar flame speeds were reviewed for agreement with experimental data, which is a standard format for validating kinetic models. Kinetic models by Glarborg from 2018[29] and 2022[30], Mei[28], Klippenstein[31], and Okafor[32] were analyzed.

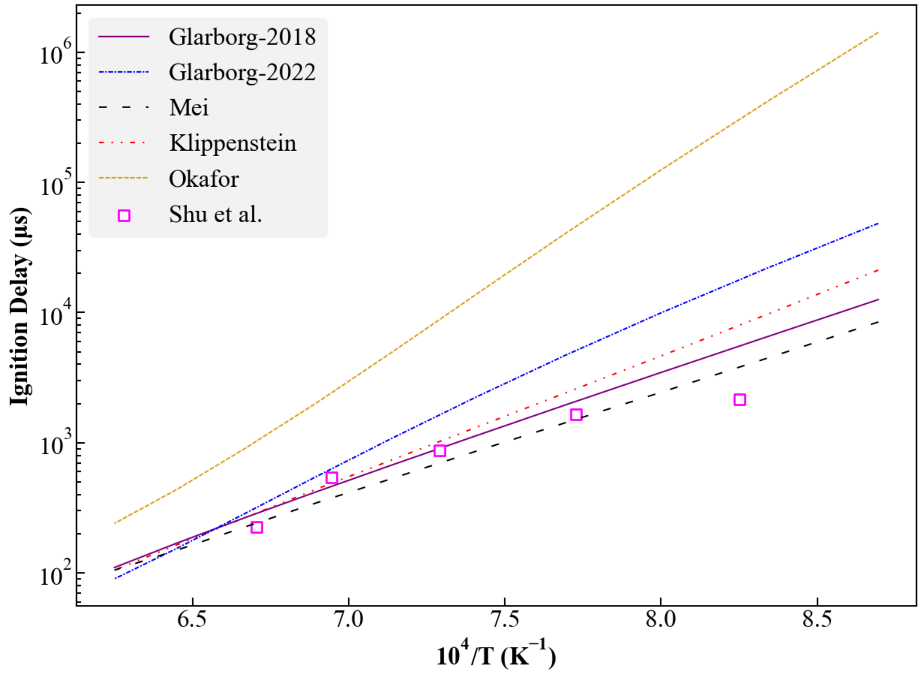

Figure S1. Comparison of ignition delay times from various reaction mechanisms with experimental data (Φ = 0.5, P = 20 bar) [adapted from Ref. 24, 2023].

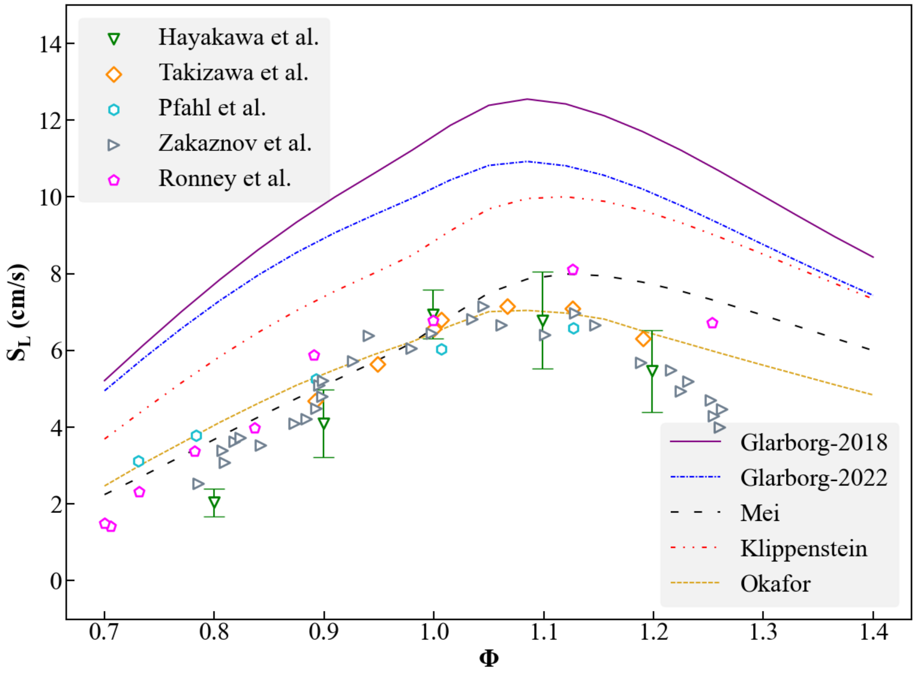

Figure S2. Comparison of laminar flame speeds from various experimental datasets and reaction mechanisms (P = 1 bar) [adapted from Ref. 24, 2023].

For ignition delay times, comparisons were done with data from a study done by Mathieu and Petersen[33]. Shu et al. [34] also published ignition delay times for ammonia-air mixtures, for which a sample comparison can be seen in Fig. S1. Laminar flame speeds over a range of equivalence ratios were compared to datasets from Hayakawa et al. [35], Takizawa et al. [36], Pfahl et al. [37], Zakaznov et al. [38], and Ronney et al. [39] (Fig. S2). Since the Mei model showed overall good

agreement with experimental data for both ignition delay and laminar flame speed, it was chosen as the suitable mechanism for this study.

REFERENCES

(26) Goodwin, D. G.; Moffat, H. K.; Schoegl, I.; Speth, R. L.; Weber, B. W. Cantera: An Object-oriented Software Toolkit for Chemical Kinetics, Thermodynamics, and Transport Processes. **2022**, *39* (1), 1415-1423. DOI: 10.5281/zenodo.6387882 (acccessed December 27, 2022).

(27) Alnasif, A.; Mashruk, S.; Kovaleva, M.; Wang, P.; Valera-Medina, A. Experimental and numerical analyses of nitrogen oxides formation in a high ammonia-low hydrogen blend using a tangential swirl burner. *Carbon Neutrality* **2022**, *1* (1), 24. DOI: 10.1007/s43979-022-00021-9.

(28) Mei, B.; Zhang, X.; Ma, S.; Cui, M.; Guo, H.; Cao, Z.; Li, Y. Experimental and kinetic modeling investigation on the laminar flame propagation of ammonia under oxygen enrichment and elevated pressure conditions. *Combustion and Flame* **2019**, *210*, 236-246. DOI: https://doi.org/10.1016/j.combustflame.2019.08.033.

(29) Glarborg, P.; Miller, J. A.; Ruscic, B.; Klippenstein, S. J. Modeling nitrogen chemistry in combustion. *Progress in Energy and Combustion Science* **2018**, *67*, 31-68. DOI: https://doi.org/10.1016/j.pecs.2018.01.002.

(30) Glarborg, P. The NH3/NO2/O2 system: Constraining key steps in ammonia ignition and N2O formation. *Combustion and Flame* **2022**, 112311. DOI: https://doi.org/10.1016/j.combustflame.2022.112311.

(31) Klippenstein, S. J.; Harding, L. B.; Glarborg, P.; Miller, J. A. The role of NNH in NO formation and control. *Combustion and Flame* **2011**, *158* (4), 774-789. DOI: https://doi.org/10.1016/j.combustflame.2010.12.013.

(32) Okafor, E. C.; Naito, Y.; Colson, S.; Ichikawa, A.; Kudo, T.; Hayakawa, A.; Kobayashi, H. Measurement and modelling of the laminar burning velocity of methane-ammonia-air flames at high pressures using a reduced reaction mechanism. *Combustion and Flame* **2019**, *204*, 162-175. DOI: https://doi.org/10.1016/j.combustflame.2019.03.008.

(33) Mathieu, O.; Petersen, E. L. Experimental and modeling study on the high-temperature oxidation of Ammonia and related NOx chemistry. *Combustion and Flame* **2015**, *162* (3), 554-570. DOI: https://doi.org/10.1016/j.combustflame.2014.08.022.

(34) Shu, B.; Vallabhuni, S. K.; He, X.; Issayev, G.; Moshammer, K.; Farooq, A.; Fernandes, R. X. A shock tube and modeling study on the autoignition properties of ammonia at intermediate temperatures. *Proceedings of the Combustion Institute* **2019**, *37* (1), 205-211. DOI: https://doi.org/10.1016/j.proci.2018.07.074.

(35) Hayakawa, A.; Goto, T.; Mimoto, R.; Arakawa, Y.; Kudo, T.; Kobayashi, H. Laminar burning velocity and Markstein length of ammonia/air premixed flames at various pressures. *Fuel* **2015**, *159*, 98-106. DOI: https://doi.org/10.1016/j.fuel.2015.06.070.

(36) Takizawa, K.; Takahashi, A.; Tokuhashi, K.; Kondo, S.; Sekiya, A. Burning velocity measurements of nitrogen-containing compounds. *Journal of Hazardous Materials* **2008**, *155* (1), 144-152. DOI: https://doi.org/10.1016/j.jhazmat.2007.11.089.

(37) Pfahl, U. J.; Ross, M. C.; Shepherd, J. E.; Pasamehmetoglu, K. O.; Unal, C. Flammability limits, ignition energy, and flame speeds in H2–CH4–NH3–N2O–O2–N2 mixtures. *Combustion and Flame* **2000**, *123* (1), 140-158. DOI: https://doi.org/10.1016/S0010-2180(00)00152-8.

(38) Zakaznov, V. F.; Kursheva, L. A.; Fedina, Z. I. Determination of normal flame velocity and critical diameter of flame extinction in ammonia-air mixture. *Combustion, Explosion and Shock Waves* **1978**, *14* (6), 710-713. DOI: 10.1007/BF00786097.

(39) Ronney, P. D. Effect of Chemistry and Transport Properties on Near-Limit Flames at Microgravity. *Combustion Science and Technology* **1988**, *59* (1-3), 123-141. DOI: 10.1080/00102208808947092.
